# Emerging Technologies in Cardiac Pacing

**DOI:** 10.1146/annurev-med-051022-042616

**Published:** 2023-11-21

**Authors:** Ramya Vajapey, Mina K. Chung

**Affiliations:** Department of Cardiovascular Medicine, Heart, Vascular & Thoracic Institute, Cleveland Clinic, Cleveland, Ohio, USA

**Keywords:** pacemaker, conduction system pacing, leadless pacing, His bundle pacing, left bundle pacing, cardiac resynchronization therapy

## Abstract

Cardiac pacing to treat bradyarrhythmias has evolved in recent decades. Recognition that a substantial proportion of pacemaker-dependent patients can develop heart failure due to electrical and mechanical dyssynchrony from traditional right ventricular apical pacing has led to development of more physiologic pacing methods that better mimic normal cardiac conduction and provide synchronized ventricular contraction. Conventional biventricular pacing has been shown to benefit patients with heart failure and conduction system disease but can be limited by scarring and fibrosis. His bundle pacing and left bundle branch area pacing are novel techniques that can provide more physiologic ventricular activation as an alternative to conventional or biventricular pacing. Leadless pacing has emerged as another alternative pacing technique to overcome limitations in conventional transvenous pacemaker systems. Our objective is to review the evolution of cardiac pacing and explore these new advances in pacing strategies.

## INTRODUCTION

**Figure 1:**
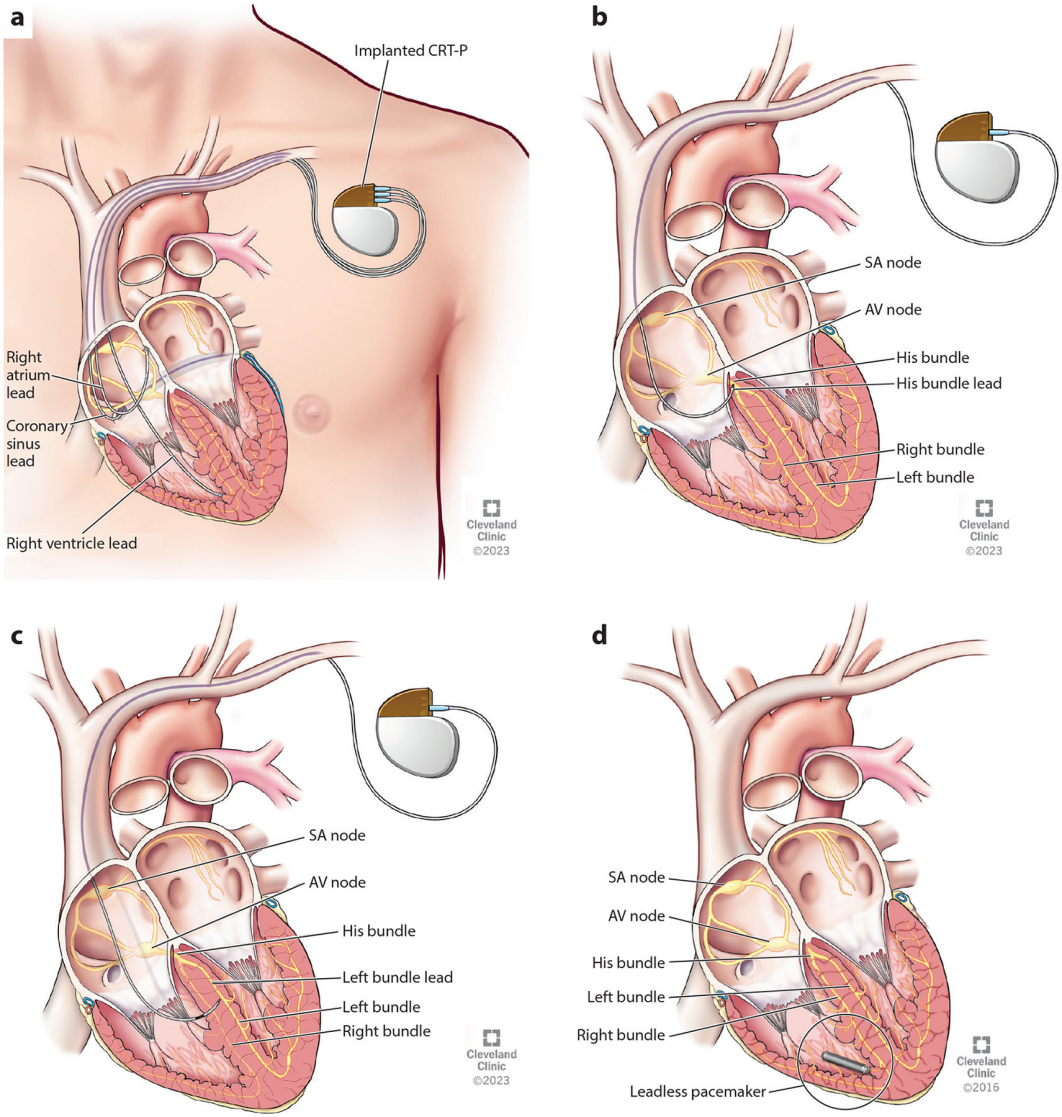
Types of cardiac pacing: (*a*) CRT-P device, (*b*) His bundle lead, (*c*) left bundle branch area pacing, (*d*) leadless pacing. Abbreviations: AV, atrioventricular; CRT-P, cardiac resynchronization therapy pacing; SA, sinoatrial. Figure reprinted with permission, Cleveland Clinic Foundation ©2023. All rights reserved.

Cardiac pacing has been the main treatment for patients with bradycardia due to various pathologies, such as sinus node dysfunction or atrioventricular (AV) block. Technological advances have been geared toward determining the optimal ventricular pacing site to best mimic normal human ventricular physiology and conduction. Long-term right ventricular (RV) pacing (RVP) can lead to pacing-induced cardiomyopathy due to left ventricular (LV) mechanical dyssynchrony in 5.9–39% of patients (1). This risk has motivated the emergence of new pacing strategies for more physiologic pacing (Figure 1a-c). These in turn have also been applied to patients with heart failure (HF) with dyssynchronous LV contraction related to conduction system disease. Conventional pacing systems are also limited by the use of transvenous leads, as well as subcutaneously placed pulse generators that can be prone to infection, vascular access challenges, and mechanical stressors that can cause lead malfunction. Leadless pacing systems have been developed (Figure 1d), which are progressing from single to dual chamber pacing capability and can now be integrated with defibrillation and transvenous components.

## CARDIAC PHYSIOLOGIC PACING

RVP was the initial pacing strategy for those requiring permanent pacemakers. However, since its development, studies have shown that RVP produces nonphysiologic LV activation with creation of left bundle branch block (LBBB)-like activation. Retrospective analysis of the Mode Selection Trial (MOST) reported that the risk of HF hospitalization and atrial fibrillation significantly increased with higher pacing burden irrespective of pacing mode (2). The Dual Chamber and VVI Implantable Defibrillator (DAVID) trial highlighted the negative consequences of ventricular dyssynchrony with RVP in patients with reduced LV systolic function. One-year survival free of the composite endpoint was 84% for patients treated with ventricular backup pacing at 40 beats/min compared with 73% for patients with dual-chamber rate-responsive pacing at 70 beats/min, with higher rates of mortality and HF hospitalization in the dual-chamber rate-responsive pacing group (3). The DAVID trial contributed to wide adoption of RVP avoidance algorithms in patients who are not dependent on ventricular pacing. In addition to reduction of unnecessary pacing, clinical practice has sought alternative strategies for more physiologic pacing to improve LV dyssynchrony and clinical outcomes. These have included cardiac resynchronization therapy (CRT) and more recently conduction system pacing (CSP), which includes His bundle pacing (HBP) and left bundle branch area pacing (LBBAP).

### Cardiac Resynchronization Therapy

CRT has emerged as one of the most remarkable advances in HF therapy and management. Biventricular pacing (BiVP) with short atrioventricular delay was proposed as a supplementary treatment of advanced HF in the 1990s (4, 5). Conduction disease, including LBBB or intraventricular conduction delay, is present in a significant proportion of patients with HF, leading to dyssynchronous ventricular contraction. CRT is typically applied with BiVP, most commonly with a RV endocardial lead and LV pacing delivered via a lead in a LV branch of the coronary sinus or less commonly a lead placed on the LV epicardium. CRT has been shown to improve functional status and improve survival outcomes in selected patients with cardiomyopathy and widened QRS durations reflective of LBBB or intraventricular conduction delay (6). Over the past few decades, the evaluation of CRT has progressed rapidly from case studies to randomized controlled trials (Table 1).

Multisite Stimulation in Cardiomyopathy (MUSTIC) was the first randomized controlled study on CRT. It showed that BiVP significantly improves exercise tolerance and quality of life in patients with chronic HF and intraventricular conduction delay. Overall, 48 patients who had severe HF [New York Heart Association (NYHA) class III] with normal sinus rhythm and QRS duration of more than 150 ms were enrolled. They underwent CRT placement with leads in the right atrium and each ventricle with a 3-month period of inactive pacing and a 3-month period of active pacing. The primary endpoint, distance walked in 6 min, was significantly greater with active pacing; secondary endpoints of quality of life measured by questionnaire, peak oxygen consumption, and hospitalizations for HF were also superior in the active pacing group (7). Subsequently, the Multicenter InSync Randomized Clinical Evaluation (MIRACLE) study was published, with blinded assessment of the effects of CRT, including symptoms, quality of life, and HF status. Similar to the MUSTIC trial, MIRACLE showed significant clinical improvement in patients who underwent CRT pacing, when randomized to blinded BiVP “on” versus “off” for 6 months (8).

The paradigm shift to use of CRT in selected populations of patients with HF was solidified after large randomized clinical trials demonstrated improvements in primary endpoints of all-cause mortality or HF hospitalizations. In the Cardiac Resynchronization—Heart Failure (CARE HF) and Comparison of Medical Therapy, Pacing, and Defibrillation in Heart Failure (COMPANION) trials (9, 10), patients with NYHA class III and IV HF due to LV systolic dysfunction and ventricular dyssynchrony with QRS duration >120 ms on optimal guideline-directed medical therapy were enrolled. CARE HF evaluated the role of CRT pacing (CRT-P), whereas COMPANION investigated CRT with a defibrillator (CRT-D), with both endpoints of death from any cause and unplanned hospitalizations for HF. These trials showed that CRT decreases HF hospitalizations and overall mortality (9, 10). Technical developments that have improved delivery of CRT include multipolar leads, which enable delivery of LV pacing from different electrode positions, and combinations, which can be advantageous in avoiding diaphragmatic stimulation and achieving more basal stimulation.

**Figure 2:**
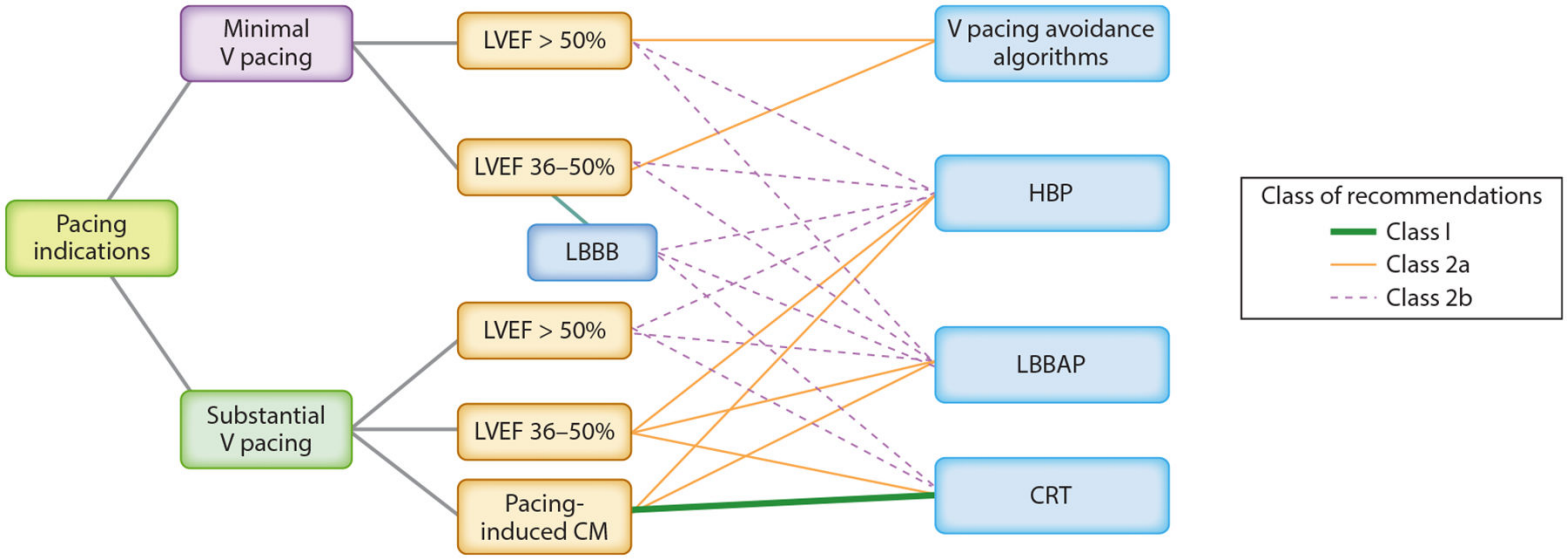
Cardiac physiologic pacing strategies for patients with pacing indications. Adapted from Reference 1 with permission. Abbreviations: CM, cardiomyopathy; CRT, cardiac resynchronization therapy; HBP, His bundle pacing; LBBAP, left bundle branch area pacing; LBBB, left bundle branch block; LVEF, left ventricular ejection fraction; V, ventricular.

**Figure 3:**
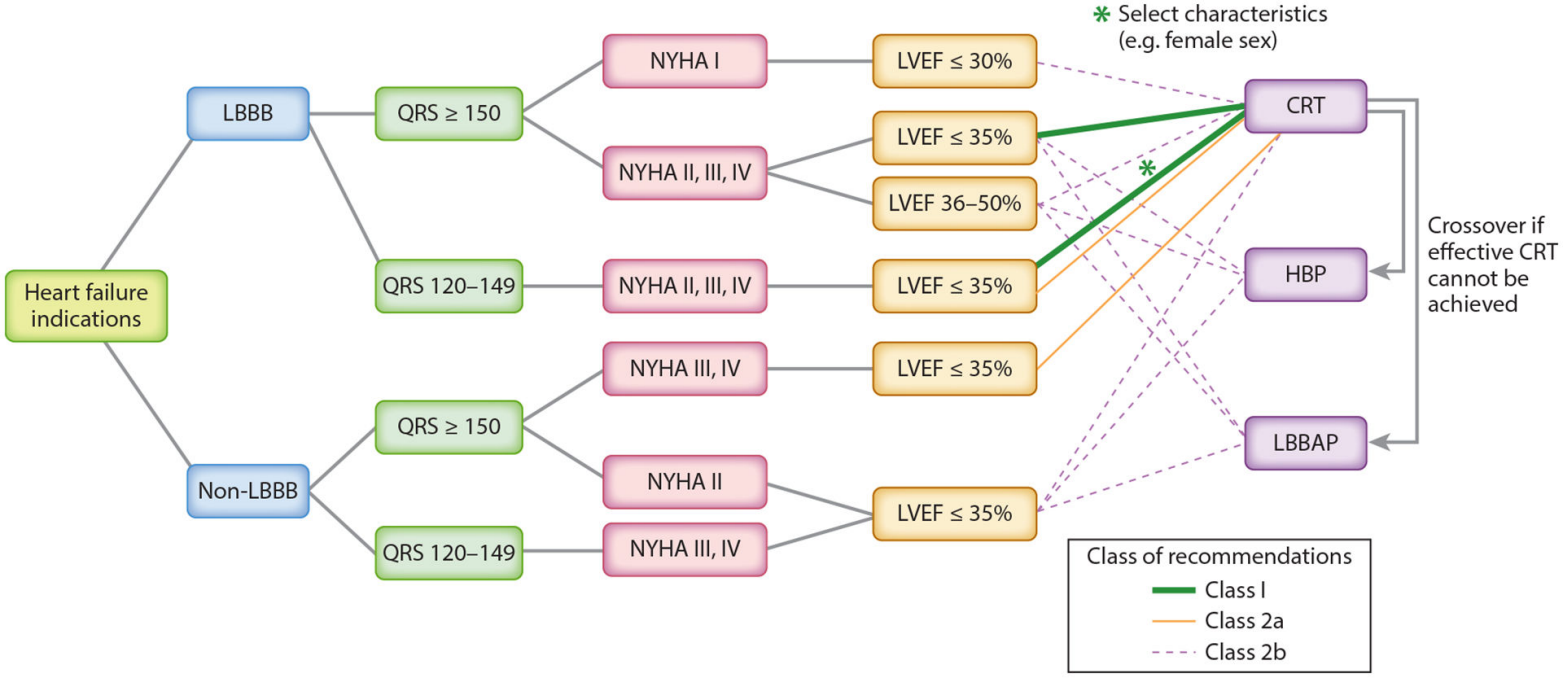
Cardiac physiologic pacing strategies for patients with heart failure indications. Adapted from Reference 1 with permission. Abbreviations: CRT, cardiac resynchronization therapy; HBP, His bundle pacing; LBBAP, left bundle branch area pacing; LBBB, left bundle branch block; LVEF, left ventricular ejection fraction; NYHA, New York Heart Association functional class.

The European Society of Cardiology and American College of Cardiology/American Heart Association HF guidelines, as modified in 2013, assigned a class I recommendation for CRT in patients with LV ejection fraction (LVEF) ≤35%, LBBB, QRS duration ≥150 ms, and NYHA functional class III or ambulatory class IV chronic HF despite adequate medical treatment (11, 12). The 2023 Heart Rhythm Society (HRS)/Asia Pacific Heart Rhythm Society (APHRS)/Latin American Heart Rhythm Society (LAHRS) Guideline on Cardiac Physiologic Pacing for the Avoidance and Mitigation of Heart Failure (1) extended class II indications for CRT to select patients with narrower QRS durations or LVEF 36–50% (Figures 2 and 3) (1).

### Conduction System Pacing: His Bundle Pacing

About 30% of patients who undergo implantation of a CRT device do not improve their HF symptoms, and the inability to stimulate severely diseased myocardium or myocardial scar without a large stimulus to QRS latency is a major obstacle for CRT delivery (13). CSP that can reduce intraventricular and atrioventricular dyssynchrony by providing a more physiologic pattern of ventricular electrical activation has gained popularity over the past few years and has emerged as a potential alternative to RVP and in some situations CRT.

The His-Purkinje system is essential for the maintenance of the synchronous ventricular contraction via endocardial to epicardial and apical to basal electrical activation. HBP emerged as an alternative to BiVP with the goal of maintaining a physiologic pattern of ventricular activation via the native His-Purkinje system (14). The His bundle lies within the membranous portion of the interventricular septum surrounded by fibrous connective tissue and divides to form right and left bundles as it enters the muscular septum (15). Initial studies were conducted in animal models (16, 17) and first described in patients in early 2000 by Deshmukh et al., who demonstrated that implanting a transvenous pacing lead to capture the His bundle has the potential to improve cardiac function and LV dimensions compared to RVP (18).

With HBP, an active fixation pacemaker lead is affixed into the proximal intraventricular septum (Figure 1b). There can be selective capture of the His bundle, in which only the His bundle is stimulated, or nonselective capture, involving fused capture of the His bundle and adjacent ventricular tissues (19). Selective capture criteria include (*a*) pacing stimulus to QRS onset interval equal to native His to QRS onset interval, (*b*) discrete local ventricular electrogram on the pacing lead, (*c*) paced QRS morphology matching native QRS morphology, and (*d*) single capture threshold (His capture). Nonselective capture criteria include (*a*) pacing stimulus to QRS onset interval usually zero, as there is no isoelectric interval between pacing stimulus and QRS, which can lead to an appearance of a pseudodelta wave from local myocardial capture; (*b*) local ventricular electrogram directly captured by pacing stimulus without a discrete component; (*c*) paced QRS duration longer than native QRS duration with concordant paced QRS electrical axis and intrinsic QRS axis; and (*d*) two distinct capture thresholds—RV and His capture (20). Selective capture results in greater reduction in QRS width; however, nonselective capture appears to have a similar LV activation time and pattern (21).

#### HBP for patients needing a pacemaker.

Retrospective and prospective studies report overall success rates of 65–94% for HBP used in patients with pacemaker indications (Table 2) (22). Pastore et al. sought to study the effects of HBP on left atrial (LA) function in patients with normal cardiac function who needed permanent pacemaker implantation (23). Patients first underwent 3 months of His area pacing followed by 3 months of RVP with various echo parameters such as systole-diastole (S-D) LV electromechanical delay, S-D intra-LV dyssynchrony, myocardial performance index and mitral annular tissue Doppler early diastolic velocity (E′), and LA function evaluated after each 3-month period. His area pacing showed more physiologic LV electromechanical activation/relaxation with higher LA volumes pre–atrial contraction and improved total emptying fraction and lower risk of atrial fibrillation progression compared to RVP (23). Another large retrospective observational study examined clinical outcomes of HBP at one hospital versus RVP at a sister hospital (24). HBP was successful in 92% of patients. The primary composite endpoint of death, HF hospitalizations, or upgrade to BiVP was significantly lower in the HBP group; this difference was primarily observed in patients with ventricular pacing burden >20% (24). Another multicenter study of 844 patients receiving HBP for pacemaker indications demonstrated reasonable long-term success rates but highlighted high pacing thresholds as a limitation of HBP, which necessitated device replacement due to battery depletion in 19.6% of patients over a mean follow-up of 5.9 years and led to interruption of HBP in 7.6% (25).

#### HBP as an alternative to CRT.

Several retrospective and prospective observational studies have used HBP in cases where CRT was indicated but where resynchronization via the coronary sinus was not achievable (26). Success rates range from 56% to 95% with significant improvements reported in QRS duration, LVEF, and NYHA functional class (Table 2). A retrospective multicenter study of 106 patients using HBP for resynchronization therapy reported that HBP was able to narrow the QRS in those with LBBB with improvement in LVEF and NYHA functional class (27). In this study, HBP was attempted as a rescue strategy in patients with failed LV lead or nonresponse to BiVP, or as a primary strategy in patients with AV block, bundle branch block, or high ventricular pacing burden as an alternative to BiVP (27).

In light of high nonresponse rates for traditional CRT, a few small randomized clinical trials have evaluated HBP in patients with CRT indications. His Pacing versus Biventricular Pacing in Symptomatic HF Patients with Left Bundle Branch Block (His-Alternative) was a randomized trial comparing two ways of achieving cardiac resynchronization (28). Fifty patients with LVEF ≤35% with LBBB and NYHA class II–IV were randomized to His-CRT versus biventricular CRT (BiV-CRT). For HF patients with LBBB, His-CRT provided similar clinical and physical improvement, including NYHA class improvement, higher LVEF, and lower LV end systolic volume compared to BiV-CRT at the expense of higher pacing thresholds (28). The His-SYNC (His Bundle Pacing versus Coronary Sinus Pacing for Cardiac Resynchronization Therapy) trial was a multicenter randomized controlled trial where patients eligible for conventional CRT were randomized to His-CRT or BiV-CRT with coronary sinus lead placement (29). Primary outcomes were reduction in QRS duration, absolute improvement in LVEF and echocardiographic response (LVEF improvement >5%) at 6 months, and cardiovascular hospitalization or death at 12 months. With a small sample size of 41 patients, study outcomes showed no significant differences in QRS duration between the two groups, similar improvement in LVEF, and no significant differences in cardiac hospitalization or death. HBP patients had higher pacing thresholds compared with BiV-CRT. This trial showed that HBP was not superior to CRT; however, it suggested HBP could be a reasonable alternative for patients undergoing CRT where coronary sinus anatomy is unfavorable (29). HOPE-HF (His Optimized Pacing Evaluated for Heart Failure trial) (30) was a multicenter prospective randomized double-blinded crossover study in 167 patients with LVEF ≤40%, PR ≥ 200 ms, and either QRS duration ≤140 s or right bundle branch block (RBBB). Patients had His bundle leads implanted and were randomized to 6 months of pacing and 6 months of no pacing in a crossover design. Neither the primary endpoint of peak oxygen uptake during symptom-limited exercise nor LVEF was increased with HBP, but quality of life improved significantly with HBP. Combined HBP with BiVP, known as His-optimized CRT (HOT-CRT), was tested in a small feasibility study of 27 patients with indications for CRT; this study reported significantly reduced paced QRS duration compared to BiVP alone and improvements in LVEF, LV volumes, and NYHA functional class (31).

In patients with conduction system disease, the success of HBP depends on the location of disease within the His-Purkinje system and, in patients with LBBB and HF, whether HBP can correct the bundle branch block. A study by Upadhyay et al. (32) showed that among 72 patients with a LBBB pattern, complete conduction block within the proximal left conduction system was observed in 64% of patients and intact Purkinje activation was seen in 36%. HBP corrected wide QRS in 85% of those with complete conduction block [both left intrahisian and proximal left bundle branch (LBB)], whereas no patients with intact Purkinje activation demonstrated correction of QRS with HBP. Disease localized to left intrahisian block was most amenable to corrective HBP by recruitment of latent Purkinje fibers (32). Intracardiac data might be more useful in predicting response to HBP than surface electrocardiography.

A limitation of HBP is its higher pacing thresholds compared to conventional RVP leads, which may lead to lower pacemaker battery longevity, need for lead revision, and/or need for placement of a back-up ventricular lead in patients who are pacemaker dependent.

### Conduction System Pacing: Left Bundle Branch Area Pacing

In patients with prolonged His-ventricular interval or LBBB, HBP may not correct left bundle conduction time because of more distal disease, in which case LBBAP may be more useful. LBBAP (Figure 1c) is a newer technique, with a limited number of studies reporting clinical outcomes and relatively short-term follow-up. Nevertheless, LBBAP leads can be easier to place than HBP leads due to the wider target areas, and pacing thresholds are generally excellent, overcoming some of the limitations of HBP. Achieving effective LBBAP with capture of the LBB or its fascicles or capture of the left septum remains important to demonstrate (33).

The initial technique of LBBAP was developed by Huang et al. (34) in 2017. The lead tip is advanced into the deep septum from the RV septal endocardium to the subendocardial LV septum to directly stimulate the left bundle area. Optimal sites of fixation are generally 1–1.5 cm distal to the His bundle. Paced QRS morphology changes from LBBB toward a RBBB pattern as the lead is advanced into the septum (34, 35). LBBAP capture can be confirmed with various criteria, which include (*a*) paced QRS morphology that shows an r′ pattern in the V1 lead, (*b*) peak LV activation time measured from onset of pacing spike to peak R wave in leads V5–6 < 80 ms, (*c*) LBB potential with sharp high-frequency deflection distance 15–30 ms to onset of surface QRS, (*d*) retrograde His or anterograde distal LBB potentials, (*e*) demonstration of differential septal and LBB capture thresholds, potentially with programmed stimulation, and (*f*) differential thresholds of selective LBBAP capturing only LBB or nonselective LBBAP capturing both LBB and adjacent local septal myocardium (36).

#### LBBAP in patients with pacing indications.

Retrospective or prospective observational studies in patients with pacemaker or CRT indications report success rates of 85–97% for LBBAP with improvements in QRS duration, LV synchrony, NYHA functional class, LVEF, and LV dimensions (Table 3) (37). Long-term safety and feasibility of LBBAP were demonstrated by a prospective study where LBBAP was attempted in 632 patients with a 97.8% success rate. At 2-year follow-up, patients had low lead complication rates, which were defined as a rise in LBB threshold >3V or loss of capture, lead dislodgments, or right bundle branch injury. Patients with LBBB had significant decreases in QRS duration, and postimplant LVEF improved in patients with QRS >120 ms (38). In the Geisinger-Rush CSP registry, 703 patients undergoing pacemaker implantation for bradycardia were divided into LBBAP (*n* = 321) and RVP groups (*n* = 382) with a primary outcome of composite all-cause mortality, HF hospitalization, or upgrade to BiVP. LBBAP compared to RVP was associated with lower composite outcomes of mortality and HF hospitalizations among patients with pacing burden >20% (39). LBBAP was evaluated in patients with HF with persistent atrial fibrillation undergoing AV junctional ablation, showing a decrease in HF hospitalizations, death, and inappropriate implantable cardioverter-defibrillator therapies (40). However, randomized trials remain limited to small feasibility studies.

#### LBBAP as an alternative to CRT.

LBBAP may be comparable or even preferable to CRT in some patients with its ability to correct LBBB and reverse LV dyssynchrony. There have been a few observational studies involving CRT-eligible patients who had LBBAP (Table 3). LBBAP in patients with HF with LBBB achieved reduction in QRS duration, shortened LV activation time, and improved LVEF and LV dimensions (41, 42). In comparison to BiVP, NYHA class, plasma N-terminal pro-brain natriuretic peptide (NTproBNP) levels, and LV size and function were significantly improved. LBBAP achieved a greater increase in LVEF compared to BiVP, with much lower pacing threshold and higher sensing than with HBP (43, 44).

Recent small studies suggesting potential benefit of LBBAP for patients in need of CRT include Left Bundle Branch Pacing Versus Biventricular Pacing in Cardiac Resynchronization Therapy: A Randomized Controlled Pilot Trial (LBBP-RESYNC Trial). This was a prospective study of nonischemic cardiomyopathy patients with LBBB randomized to LBBAP or BiVP with 6-month follow-up. The primary endpoint was difference in LVEF improvement between the two groups. The LBBAP-CRT group demonstrated greater LVEF improvement (mean difference 5.6%) than the BiVP-CRT group with improvement in LV end-systolic volume, NTproBNP level, NYHA class, and 6-min walk distance (45).

Recent studies have also demonstrated LBBAP as a viable alternative option for CRT in patients who failed traditional BiVP due to coronary sinus lead implantation failure or failure to respond to BiVP (46). CSP with HBP or LBBAP compared to BiVP was studied in an observational nonrandomized cohort of 477 patients with class I or II indications for CRT; the investigators reported that CSP was associated with significantly lower mortality and HF hospitalization (47). The Left Bundle Branch-Optimized CRT (LOT-CRT) study, from an international LBBAP collaborative group, assessed LBBAP-optimized CRT combined with a coronary venous LV pacing lead with conventional BiVP [RV and coronary sinus (CS) leads] versus LBBAP alone (without a CS lead). LOT-CRT was associated with a narrower QRS duration than BiVP or LBBAP alone and showed improved LVEF, LV volume, and NYHA class compared to baseline. The authors concluded that LOT-CRT is a feasible option and could be an alternative when suboptimal electrical resynchronization is obtained with BiV-CRT (48). Pivotal randomized clinical trials for CSP compared to CRT in patients meeting indications for CRT are ongoing or planned. These include the Left versus Left Randomized Clinical Trial (NCT05650658), which will compare His or LBB pacing to BiVP in >2,000 patients with HF (LVEF ≤ 50%) and either with a wide QRS (≥130 ms) or with/anticipated >40% pacing. The need for continued assessment of long-term outcomes was highlighted by a European MELOS registry of LBBAP outcomes in 2,533 patients that reported an 8.3% incidence of complications specific to the ventricular transseptal pacing route (49).

Though LBBAP has been the latest approach introduced for cardiac physiologic pacing, LBBAP has been emerging as a dominant approach over HBP due to its superior technical performance with lower pacing thresholds, better sensing, and larger implant target. Recommendations for CSP, including HBP and LBBAP, are reported in the 2023 HRS/APHRS/LAHRS Guideline on Cardiac Physiologic Pacing for the Avoidance and Mitigation of Heart Failure and are summarized in Figures 1 and 2 (1).

## LEADLESS PACING

Leadless pacemakers were recently introduced to address lead- and pocket-related complications in conventional transvenous pacemaker systems. Transvenous leads can cause complications, such as venous obstruction, tricuspid regurgitation, and endocarditis, with transvenous lead-related endocarditis mortality rates up to 12–31% (50-52). Pocket-related complications can include skin erosion, pocket infection, and potential sepsis (53, 54). To address these issues with transvenous pacemaker systems, a leadless pacemaker was initially conceptualized in the 1970s (55) and implanted in 2014 (56). The first approved devices are single-component systems that can provide single-chamber RVP, sensing, and delivery of rate response. One of the single-chamber devices has an algorithm to sense atrial contractions, enabling a degree of AV synchronous pacing. Leadless pacemakers are implanted via a percutaneous femoral catheter-based approach to advance the pacemaker into the RV.

One of the first trials in leadless pacing enrolled 33 patients at three centers to evaluate clinical safety and performance of a RV leadless pacing system. The device was successfully implanted in 97% of patients with overall complication-free rate of 94%, adequate electrical performance, and no device-related complications at 12-month follow-up (56, 57). A second clinical study enrolled 526 patients with implantation success rate of 96%, with device-related complications (such as pericardial effusion, vascular complications, and device dislodgment) reported in 6.4% of patients (58). At 12-month follow-up, the complication rate was 71% lower than that of transvenous systems (59). Using another RV leadless device, another study enrolled 725 patients; the device was successfully implanted in 99% of patients, with major complications such as cardiac injury and vascular access issues occurring in 4% of patients (60). Short-term complications of leadless pacemakers and transvenous pacemaker systems were comparable (61). Long-term, 12-month follow-up of leadless pacemakers compared to propensity-matched transvenous system controls showed a 48% lower risk of major complications and 82% lower occurrence of revisions or reinterventions (62, 63). Longer-term outcome data are needed for leadless pacemakers, as at the end of service, either devices are extracted and replaced, if possible, or an additional device(s) is placed. A dual-chamber leadless pacemaker system was recently approved by the FDA.

Leadless LV endocardial pacing has also been studied for CRT in prospective multicenter observational studies (63, 64). One such system consists of a subcutaneous ultrasound transmitter/pulse generator and a rice-sized receiver electrode implanted in the LV endocardium. Ultrasonic energy is delivered to the LV endocardial receiver electrode to achieve BiVP. Studies have included patients who failed conventional CRT due to failed CS lead cannulation or non-responders to traditional CRT. However, significant complications include cardioembolic stroke, pericardial effusion, catheter-induced ventricular fibrillation, electrode embolization, and femoral artery access issues (65-70).

## FUTURE DIRECTIONS

Conventional CRT has evolved over the years into a highly effective therapy for HF patients with LBBB. CSP has emerged as an alternative pacing strategy for those patients in whom conventional CRT does not produce satisfactory clinical response, when there is difficulty with CS lead placement, or as an alternative to RVP. Recent studies have shown comparable and in some cases more effective electrical and mechanical resynchronization with CSP with HBP or LBBAP compared to BiVP, although the evidence base for CRT includes randomized controlled trials showing improved survival and reduction in HF hospitalizations in selected patients with HF and widened QRS duration, particularly with LBBB and QRS duration ≥150 ms. Although CSP reestablishes physiologic ventricular activation, it has lagged the evidence base of CRT with a lack of large randomized controlled trial data. However, large randomized clinical trials are launching and should provide guidance as to the suitability of CSP for HF patients. Leadless pacemakers have emerged recently with promising efficacy and safety and could be potentially used for dual-chamber pacing or resynchronization therapy. Leadless cardiac pacemakers may potentially transform cardiac pacing; however, long-term data are needed to verify device safety, performance, and extractability.

## Figures and Tables

**Table 1:** Major clinical trials of cardiac resynchronization therapy

Study	Year	Sample size	Endpoint	Design	Findings
MUSTIC-SR (7)	2001	48	6MWT, QOL, pVO_2_, Hosp	Single-blinded, randomized controlled, crossover, 6 months	CRT-P improved 6MWT, QOL, pVO_2_; reduced Hosp
MIRACLE (8)	2002	453	NYHA, QOL, pVO_2_	Prospective, randomized, double-blind, parallel controlled	CRT-P improved NYHA, pVO_2_, 6MWT
MUSTIC-AF (71)	2002	131	6MWT, QOL, pVO_2_, Hosp	Single-blinded, controlled, crossover, 6 months	CRT-P (high dropout rate) improved all; reduced Hosp
PATH-CHF (64)	2002	42	6MWT, pVO_2_	Single-blinded, randomized, crossover controlled	CRT-P improved 6MWT, pVO_2_
MIRACLE-ICD (72)	2003	369	6MWT, QOL, Hosp	Prospective, multicenter, randomized, double-blind, parallel controlled	CRT-D improved all compared to baseline
MIRACLE-ICD II (73)	2004	186	VE/CO_2_, pVO_2_, NYHA, QOL, 6MWT, LV volumes/LVEF	Double-blinded, ICD versus CRT-D, 6 months	CRT-D improved NYHA, VE/CO_2_, volumes, LVEF
COMPANION (10)	2004	1,520	All-cause death or Hosp	Prospective, multicenter, randomized controlled	CRT-P/CRT-D reduced all-cause death and Hosp
CARE-HF (9)	2005	813	All-cause death or Hosp for major CV event and death from any cause	Prospective, multicenter, randomized controlled	CRT-P reduced all-cause death or Hosp from major CV event and death from any cause
REVERSE (74)	2008	610	HF clinical composite response, LVESVi, HFH, mortality	Double-blinded, randomized controlled, GDMT, CRT-P ± ICD, 12 months	No change in HF clinical composite response or mortality, reduction in LVESVi and HFH
MADIT-CRT (75)	2009	1,817	HF events or death and LVESVi	Randomized controlled, CRT-P, CRT-D, 2.4 years	CRT-D reduced HF events or death and LVESVi but no change in mortality
RAFT (76)	2010	1,798	Death from any cause or HFH, death from any cause, death from CV cause and HFH	Randomized controlled, ICD alone versus CRT-D, 40 months	CRT-D reduced death from any cause or HFH, death from any cause, and HFH

Abbreviations: 6MWT, 6-min walk test; CRT, cardiac resynchronization therapy; CRT-D, biventricular pacer with a defibrillator; CRT-P, biventricular pacemaker; CV, cardiovascular; GDMT, guideline-directed medical therapy; HF, heart failure; HFH, heart failure hospitalization; Hosp, hospitalizations; ICD, implantable cardioverter-defibrillator; LV left ventricular; LVEF, left ventricular ejection fraction; LVESVi, left ventricular end-systolic volume index; NYHA, New York Heart Association functional class; pVO_2_, peak oxygen consumption; QOL, quality of life; VE/CO_2_, ventilation/carbon dioxide ratio.

**Table 2:** Major studies of His bundle pacing

Reference	Year	Sample size	Endpoint	Design	Findings
18	2000	18	LVEDD, LVESD, LVEF, functional status	Prospective, patients with chronic AF, LVEF <40%, QRSd <120 ms	Improvement in LV dimensions, functional status, cardiothoracic ratio, LVEF
77	2006	18	NYHA, 6MWT, QOL, hemodynamic parameters	Prospective, crossover, patients with chronic AF, AV junction ablation randomized to 6 months of RVP versus para-Hisian HBP	Improvement in NYHA, 6MWT, QOL, hemodynamic parameters
78	2010	91	Not reported	Prospective, all patients with AV block as pacing indication	No long-term clinical outcomes
26	2013	16	NYHA, LVEF, LV dimensions	Prospective, HBP in patients with failed LV leads	Improvement in NYHA; improvement in LVEF and LV dimensions
79	2014	38	LVEF, NYHA, 6MWT, QOL	Prospective, crossover, randomized patients with AV block, narrow QRS, LVEF >40%, 12 months HBP versus RVP	Improvement in LVEF; no significant improvement in NYHA, 6MWT, QOL
80	2015	38	Pacing configurations	Prospective, HBP in patients with indication for CRT	Lower pacing thresholds using bipolar HBP lead and RV lead configuration
81	2015	94	HFH, mortality and AF	Prospective, patients with PPM indication, HBP versus RVP	Improvement in HFH; no significant improvement in mortality or AF
24	2018	332	All-cause mortality and HFH	Retrospective, patients with PPM indication, HBP versus RVP	Improvement in composite end point (all-cause mortality, HFH, and upgrade to BiV pacing) with HBP
27	2018	39	NYHA, LVEF, CRT response	Retrospective, multicenter, HBP in patients with RBBB, CRT indication as primary or rescue strategy	Improvement in NYHA, LVEF, overall response rate
82	2019	74	NYHA, LVEF, LV dimensions	Prospective, HBP in patients with LBBB and indication for CRT	Improvement in NYHA and LVEF; decrease in LV dimensions
83	2019	60	QRSd, LVEF	Retrospective, HBP for CRT in patients with chronic RVP	Improvement in QRSd and LVEF
29 (His-Sync Trial)	2019	41	QRSd, LVEF at 6 months, time to cardiovascular Hosp or death at 12 months	Prospective, randomized controlled trial, His-CRT versus CS BiV-CRT; crossover mandated for HBP if failed to achieve 20% QRS narrowing, QRS width ≤130 ms, or high pacing thresholds >5 V/1 ms	His-CRT is not superior to BiV-CRT electrocardiographic (QRSd) or echo (LVEF) parameters
28 (His-Alternative Trial)	2021	50	QRSd, NYHA, LVEF, LVESV	Prospective, randomized trial His-CRT versus BiV-CRT, with crossover permitted in patients with HF	His-CRT provided similar clinical and physical improvement compared to BiV-CRT at expense of higher pacing thresholds

Abbreviations: 6MWT, 6-min walk test; AF, atrial fibrillation; AV, atrioventricular; BiV, biventricular; CRT, cardiac resynchronization therapy; CS, coronary sinus; HBP, His bundle pacing; HFH, heart failure hospitalization; Hosp, hospitalizations; LBBB, left bundle branch block; LV left ventricular; LVEDD, left ventricular end-diastolic dimension; LVEF, left ventricular ejection fraction; LVESD, left ventricular end-systolic dimension; LVESV, left ventricular end-systolic volume; NYHA, New York Heart Association functional class; PPM, permanent pacemaker; QOL, quality of life; QRSd, QRS duration; RBBB, right bundle branch block; RVP, right ventricular pacing.

**Table 3:** Major studies of left bundle branch area pacing

Reference	Year	Sample size	Endpoint	Design	Findings
84	2019	85	LV mechanical synchrony (on SPECT MPI)	Retrospective, LBBAP versus HBP in patients with AV block	LV mechanical synchrony similar in HBP and LBBAP
85	2019	33	LV synchrony (echo strain analysis), QRSd	Prospective, observational in patients with AV block	LV synchrony improved at 3-month follow-up compared to baseline, improved QRSd
43	2019	11	NYHA, NTproBNP, LVESD, LVEF	Retrospective, in patients with CRT indication	Improvement in NYHA, NTproBNP, LV size and function
44	2021	137	LVEF, NYHA, pacing threshold	Observational, patients with indication for CRT assigned to LBBAP versus HBP versus BiV pacing	HBP and LBBAP had greater improvement in LVEF and NYHA than BiV pacing. Pacing threshold lower in LBBAP than HBP
86	2020	63	NYHA, LVESV, LVEF	Prospective multicenter observational study in patients with indication for CRT	Improvement in NYHA, LVESV, LVEF
41	2021	325	QRSd, LVEF, clinical (no HFH and improvement in NYHA) and echocardiographic responses (≥5% improvement in LVEF)	Retrospective, multicenter in patients with indication for CRT	Improvement in QRSd, clinical and echocardiographic response
38	2021	632	QRSd, LVEF, LBB capture threshold, lead revision	Prospective, observational study, in patients with attempted LBBAP for bradycardia or HF indication	LBBAP had high success rate with low complications during follow-up up to 24 months
87 (LBBP-RESYNC Trial)	2022	40	LVEF, LVESV, NTproBNP; NYHA, 6MWT, QRSd	Prospective, randomized, LBBAP versus BiV pacing in patients with NICM, LBBB, and NYHA II–IV for 6 months follow-up	LBBAP had better LVEF improvement, greater reduction in LVESV and NTproBNP versus BiV-CRT; NYHA, 6MWT, QRSd similar in both groups

Abbreviations: 6MWT, 6-min walk test; AF, atrial fibrillation; AV, atrioventricular; BiV, biventricular; CRT, cardiac resynchronization therapy; HBP, His bundle pacing; HF, heart failure; HFH, heart failure hospitalization; LBB, left bundle branch; LBBB, left bundle branch block; LBBAP, left bundle branch area pacing; LV, left ventricular; LVEDD, left ventricular end-diastolic dimension; LVEF, left ventricular ejection fraction; LVESD, left ventricular end-systolic dimension; LVESV, left ventricular end-systolic volume; MPI, myocardial performance index; NICM, nonischemic cardiomyopathy; NTproBNP, N-terminal pro-brain natriuretic peptide; NYHA, New York Heart Association functional class; QRSd, QRS duration; RBBB: right bundle branch block; SPECT, single photon emission computed tomography.

## Abbreviations

AV: atrioventricular
RV: right ventricular
RVP: right ventricular pacing
LV: left ventricular
HF: heart failure
LBBB: left bundle branch block
CRT: cardiac resynchronization therapy
CSP: conduction system pacing
HBP: His bundle pacing
LBBAP: left bundle branch area pacing
BiVP: biventricular pacing
LVEF: left ventricular ejection fraction
BiV-CRT: biventricular cardiac resynchronization therapy
